# Genetic model selection for a case–control study and a meta-analysis

**DOI:** 10.1016/j.mgene.2015.04.003

**Published:** 2015-05-22

**Authors:** Nobuyuki Horita, Takeshi Kaneko

**Affiliations:** Department of Pulmonology, Yokohama City University Graduate School of Medicine, Yokohama, Japan

**Keywords:** OR, odds ratio, M, Major allele, m, minor allele, MM, homozygote of major allele, Mm, heterozygote, mm, homozygote of minor allele.

## Abstract

A case–control study often compares the prevalence of a specific disease among persons with normal alleles and persons with variant alleles, which generates an odds ratio (OR). The most common type of allele variation, single-nucleotide polymorphism, consists of a major allele (M) and a minor allele (m). Thus, the genotype can be a major allele homozygote (MM), a heterozygote (Mm) or a minor allele homozygote (mm). Odds are given for each genotype, and a pair of odds generates an OR. Summarizing data using two-by-two contingency is the simplest method of estimating an OR. Thus, dominant, multiplicative, recessive, and over-dominant models are often used. Traditionally, researchers used to calculate ORs using many models and then select the best model from among these calculated ORs. This may cause problems due to multiple comparisons. Therefore, we should choose the best model before calculating the OR for each model. In this article, we will discuss how to choose the best model among many subject-level models when evaluating the impact of the MM/Mm/mm genotype on the disease prevalence.

## Highlights

• A case-control study often compares the prevalence of a specific disease among persons with normal alleles and persons with variant alleles, which generates an odds ratio (OR). Traditionally, researchers used to calculate ORs using many models and then select the best model from among these calculated ORs. In this article, we will discuss how to choose the best model among many subject-level models before calculating ORs using some models.

## Introduction

Heredity involves the passing of traits, and occasionally the risk of diseases, to offspring from their parents. This phenomenon was known long before DNA was discovered in the 20th century. Mendelian inheritance is observed for some rare diseases. On the other hand, most common diseases do not present typical Mendelian inheritance. According to the common disease–common variant hypothesis, some of those common variants lead to susceptibility to complex polygenic diseases. Each variant of each gene that influences a complex disease will have a small effect on the disease phenotype and susceptibility (Marian, 2012, Pritchard and Cox, 2002). Case–control studies, often in the form of genome-wide association studies or meta-analysis, have been conducted to discover causative variants and to evaluate the impact of gene polymorphism on a specific disease.

A case–control study often compares the prevalence of a specific disease among persons with normal alleles and persons with variant alleles, which generates an odds ratio (OR). The most common type of allele variation, single-nucleotide polymorphism, consists of a major allele (M) and a minor allele (m). Thus, the genotype can be a major allele homozygote (MM), a heterozygote (Mm) or a minor allele homozygote (mm). Odds are given for each genotype, and a pair of odds generates an OR (Table 1). Summarizing data using two-by-two contingency is the simplest method of estimating an OR. Therefore, the three kinds of genotypes are often transformed into two variables. For example, a dominant model compares MM versus Mm + mm, and a recessive model compares MM + Mm versus mm. An over-dominant model assumes the heterozygote has the strongest impact and compares MM + mm versus Mm. On the other hand, co-dominant models including additive and multiplicative models hypothesize that MM, Mm, and mm are associated with the lowest, the intermediate, and the highest risk, respectively, or they are associated with the highest, the intermediate, and the lowest risk, respectively (Thakkinstian et al., 2005, Attia et al., 2003). While these models above discuss a subject-level phenomenon, the allelic model evaluates the impact of individual alleles on the disease. This allelic model produces an OR similar to that estimated from the multiplicative model (Thakkinstian et al., 2005, Attia et al., 2003).

Traditionally, researchers used to calculate ORs using many models and then select the best model from among these calculated ORs (Thakkinstian et al., 2005, Attia et al., 2003). This may increase the possibility of type I error due to multiple comparisons (Bagos, 2013). Therefore, we should choose the best model before calculating the OR for each model. Although model selection for genome-wide study was explained by Bagos (Bagos, 2013), another method for model selection for case–control study has been anticipated. In this article, we will discuss how to choose the best model among many subject-level models when evaluating the impact of the MM/Mm/mm genotype on the disease prevalence.

## Methods and examples

In this article, for the additive model, we supposed the impact of Mm allele was estimated from the additive mean of impacts of MM and mm alleles. Similarly, for the multiplicative model, we supposed the impact of the Mm allele was estimated from the multiplicative mean of impacts of the MM and mm alleles. Although we knew that some researchers use the wording “log-additive model” instead of “multiplicative model” which is defined above and the wording “additive model” instead of “multiplicative model” which is defined above, we did not use these wordings for the current article.

### Commonly used models

The most commonly used five subject-level gene models are recessive, multiplicative, additive, dominant, and over-dominant models (Thakkinstian et al., 2005, Attia et al., 2003). Each of the five models was originally defined using the relationship among odd_MM_, odd_Mm_, and odd_mm_. Using the formulas in legends of Table 1, we can obtain the relationship between OR1 and OR2 for each model.

The recessive model is defined by odd_Mm_ = odd_MM._ Therefore, OR1 = 1.

The multiplicative model is defined by odd_Mm_ = √ (odd_MM_ × odd_mm_). Therefore, OR1 = OR2.

The additive model is defined by odd_Mm_ = (odd_MM_ + odd_mm_) / 2. Therefore, OR2 = 2 − 1 / OR1.

The dominant model is defined by odd_mm_ = odd_Mm._ Therefore, OR2 = 1.

The over-dominant model is defined by odd_mm_ = odd_MM_. Therefore, OR2 = 1 / OR1.

**Fig. 1:**
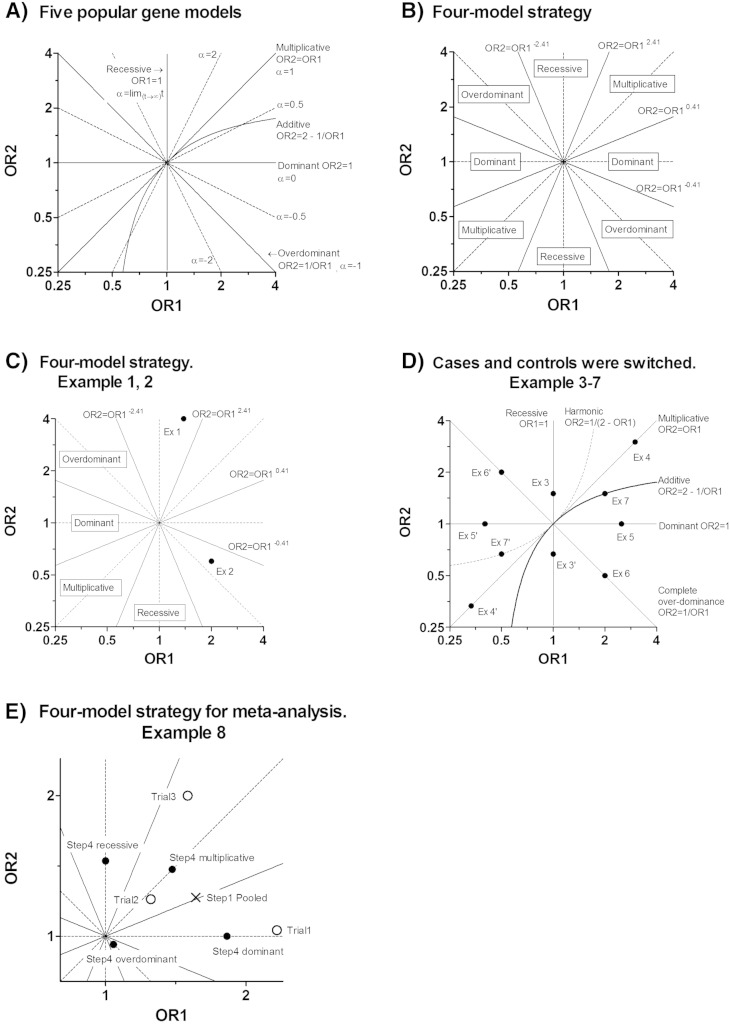
Graphical explanation of the gene models.

In the log–scale OR1–OR2 plane, the recessive, multiplicative, dominant and over-dominant models were drawn linearly, while the additive model was drawn as a curved line (Fig. 1-A). This meant that a logistic regression analysis was applicable for the first four models by applying the explanatory variables that are shown in Table 2 to persons with MM, Mm, and mm genotypes. However, it is difficult to apply logistic regression analysis to an additive model.

Data should be summarized by 2 by 3 contingency for multiplicative and additive models. Logistic regression assumes odds for a disease increases exponentially as a number of minor allele (0, 1 or 2) increases. This hypothesis exactly fits the multiplicative model but not for additive model.

### Four-model strategy

We proposed to principally use the four genetic models, i.e. the recessive, multiplicative, dominant, and over-dominant models. We selected these four models because they are the only ones that are easily applicable to logistic regression analysis, and because they are symmetrically allocated in the log–scale OR1–OR2 plane (Fig. 1-A).

Next, we defined new variables. OR1ori and OR2ori are OR1 and OR2 that are estimated from the original number of subjects observed. OR1mod and OR2mod are ORs obtained using one of the genetic models.

The first step in the four-model strategy is to calculate OR1ori and OR2ori. We can obtain OR1ori and OR2ori from a two-by-two contingency. Alternatively, we can also estimate OR1ori and OR2ori from logistic regression analysis.

The second step is to choose one optimal model among the four models. For this purpose, we draw four border lines that symmetrically divide the log–scale OR1–OR2 plane into four areas (Fig. 1-B). Borders are indicated with the following formulas: OR2 = OR1^2.41^, OR2 = OR1^0.41^, OR2 = OR1^− 0.41^, and OR2 − OR1^2.41^. Here, 0.41 and 2.41 were derived from tan ((1 / 8) × pai) and tan ((3 / 8) × pai). We select the best model by plotting (OR1ori, OR2ori) on the log–scale OR1–OR2 plane.

The third step is to calculate ORstep3, which represents a one point increase of the explanatory variables. ORstep3 can be obtained from single-variable logistic regression analysis. To apply logistic regression analysis, explanatory variables depending on the gene model indicated in Table 2 were given for each subject. Objective variables were also given: 0 for a control and 1 for a case. For the recessive, dominant, and over-dominant models, we can use a two-by-two contingency to estimate ORstep3, instead of this logistic regression analysis; though the multiplicative model always requires logistic regression.

In the fourth step, OR1mod and OR2mod are calculated from OR step3. Given the relationship between OR1 and OR2 in the previous section for each model, OR1mod and OR2mod were provided as follows: OR1mod = 1, OR2mod = ORstep3 for the recessive model; OR1mod = OR2mod = ORstep3 for the multiplicative model; OR1mod = ORstep3, OR2mod = 1 for the dominant model; OR1mod = ORstep3, OR2mod = 1 / ORstep3 for the over-dominant model.

Table 3 and Fig. 1-C present Examples 1 and 2.

### Additive and harmonic models

Usually, a diseased subject is regarded as a case, and a healthy subject is regarded as a control. However, this definition is only based on convention. Therefore, we should be able to provide an optimal model even if the number of cases and the number of controls are switched. Recessive, multiplicative, dominant and over-dominant models are still applicable, even if the numbers of cases and controls are switched. Table 4 and Fig. 1-D present Examples 3–6. However, the additive model is not applicable, once the numbers of cases and controls are switched. Table 4 and Fig. 1-D present Example 7.

We proposed to use a harmonic model that is defined by odd_Mm_ = 1 / ((1 / odd_MM_) + 1 / odd_mm_) / 2). Here, the harmonic mean, along with the multiplicative and the additive means, is a type of generalized mean. Using this relationship among odd_MM_, odd_Mm_, and odd_mm_ and the formula in the legends of Table 1, we can find out the relationship OR2 = 1 / (2-OR1). The additive model and the harmonic model are mutually exchanged when the numbers of cases and the controls are switched. Table 4 and Fig. 1-D present Example 7.

Using only an additive model but not using a harmonic model is an unfair procedure. Therefore, when considering an additive model, we also have to suppose a harmonic model simultaneously. Anyway, it is difficult to use these two models because they do not fit a logistic regression formula.

### Four-model strategy for meta-analysis

Meta-analysis is often conducted to estimate the true impact of each allele variant on susceptibility to a specific disease. For this, the four-model strategy is also applicable.

First, OR1ori and OR2ori are estimated from the number of subject in each original study. Then pooled OR1ori and pooled OR2ori are calculated.

Second, the best model plotting (pooled OR1ori, pooled OR2ori) on the log–scale OR1–OR2 plane is chosen.

Third, the ORstep3 for the selected model is estimated from the number of subjects in each original study. Then the pooled ORstep3 is calculated from ORstep3.

Fourth, OR1mod and OR2mod are calculated from the pooled ORstep3_._

Table 5 and Fig. 1-E present Example 8.

## Conclusion

The impact of allele variation on non-Mendelian diseases is investigated in many case–control studies and meta-analyses. However, an acceptable strategy to select the best model has not been developed. We developed a novel method to select the best method from among recessive, multiplicative, dominant, and over-dominant models preceding the calculation of the OR for each model.

## Financial statement

No support in the form of grants, gifts, equipment, and/or drugs was provided for the current study.

## Figures and Tables

**Table 1:** Genotypes, odds and odds ratio (OR).

	MM	Mm	mm
Number of cases	a	b	c
Number of controls	d	e	f
Odd	a/d	b/e	c/f

M: Major allele.

m: minor allele.

MM: homozygote of major allele.

Mm: heterozygote.

mm: homozygote of minor allele.

a, b, c, d, e, f: number of subjects.

We defined OR1 and OR2 as follows:OR1 = odd_Mm_ / odd_MM_ = bd / aeOR2 = odd_mm_ / odd_Mm_ = ce / bf.

Therefore, odd_Mm_ = b / e = odd_MM_ × OR1ori odd_mm_ = c / f = odd_MM_ × OR1ori × OR2ori.

**Table 2:** Explanatory variables for each genotype.

	MM	Mm	mm
*OR compared to MM*			
	1	OR1	OR1 × OR2

*Explanatory variables*			
Recessive	0	0	1
Multiplicative	0	1	2
Dominant	0	1	1
Overdominant	0	1	0

**Table 3:** Four-model strategy (Examples 1 and 2).

	Original data				First step	Second step	Third step				Forth step			
						ORstep3 (95%CI)				(OR1mod, OR2mod)			
	MM	Mm	mm	(OR1ori, OR2ori)		Re	Mu	Do	Ov	Re	Mu	Do	Ov
Example 1	Case	8	8	4	(1.38, 4.00)	Re	4.75 (1.07-21.0)	2.02 (0.96 − 4.25)	1.83 (0.68 − 4.97)	1.00 (0.37 − 2.72)	(1, 4.75)	(2.02, 2.02)	(1.83, 1)	(1.00, 1.00)
Control	44	32	4
Example 2	Case	20	80	60	(2.00, 0.60)	Ov	0.72 (0.44 − 1.18)	0.95 (0.67 − 1.35)	1.56 (0.79 − 3.05)	1.75 (1.06 − 2.88)	(1, 0.72)	(0.95, 0.95)	(1.56, 1)	(1.75, 0.57)
Control	20	40	50

MM: homozygote of major allele.

Mm: heterozygote.

mm: homozygote of minor allele.

Re: recessive model.

Mu: multiplicative model.

Do: dominant model.

Ov: over-dominant model.

For OR1ori OR2ori, ORstep3, OR1mod, and OR2mod, please see main text.

For third and fourth steps, models that were not selected in the second step were also presented for the purpose of comparison.

**Table 4:** Model selection when numbers of cases and controls are switched.

		MM	Mm	mm	OR1	OR2	Model
Example 3	Case	40	40	60	1	1.5	Recessive
Control	20	20	20	
Example 3′	Case	20	20	20	1	0.67	Recessive
Control	40	40	60	
Example 4	Case	10	60	90	3	3	Multiplicative
Control	10	20	10	
Example 4′	Case	10	20	10	0.33	0.33	Multiplicative
Control	10	60	90	
Example 5	Case	8	40	40	2.5	1	Dominant
Control	10	20	20	
Example 5′	Case	10	20	20	0.4	1	Dominant
Control	8	40	40	
Example 6	Case	10	40	20	2	0.5	Over-dominant
Control	10	20	20	
Example 6′	Case	10	20	20	0.5	2	Over-dominant
Control	10	40	20	
Example 7	Case	10	40	60	2.0	1.5	Additive
Control	10	20	20	
Example 7′	Case	10	20	20	0.5	0.67	Harmonic
Control	10	40	60	

MM: homozygote of major allele.

Mm: heterozygote.

mm: homozygote of minor allele.

**Table 5:** Four-model strategy for meta-analysis (Example 8).

	Original data				First step	Second step	Third step				Fourth step			
	MM	Mm	mm	(OR1ori, OR2ori)		ORstep3 (95%CI)				(OR1mod, OR2mod)			
							Re	Mu	Do	Ov	Re	Mu	Do	Ov
Trial 1	Case	5	70	60	(2.33, 1.03)		1.12	1.24 (0.81-1.87)	2.36	1.08				
	Control	10	60	50								
Trial 2	Case	20	50	30	(1.25, 1.20)		1.29	1.22 (0.73 − 2.06)	1.33	1.00				
	Control	10	20	10								
Trial 3	Case	20	20	20	(1.50, 2.00)		2.50	1.71 (1.45 − 4.11)	2.00	1.00				
	Control	45	30	15								
Pooled					(1.56, 1.21)	Mu	1.45 (0.90 − 2.34) Z = 1.51	1.39 (1.08-1.81) Z = 2.51	1.82 (1.13 − 2.95) Z = 2.45	1.04 (0.73 − 1.48) Z − 0.21	(1, 1.45)	(1.39, 1.39)	(1.82,1)	(1.04, 0.96)

MM: homozygote of major allele.

Mm: heterozygote.

mm: homozygote of minor allele.

Re: recessive model.

Mu: multiplicative model.

Do: dominant model.

Ov: over-dominant model.

For OR1ori OR2ori, ORstep3, OR1mod, and OR2mod, please see main text.

For third and fourth steps, models that were not selected in the second step were also presented for the purpose of comparison.

## References

[bb0020] Attia J., Thakkinstian A., D'Este C. (2003). Meta-analyses of molecular association studies: methodologic lessons for genetic epidemiology. J. Clin. Epidemiol..

[bb0025] Bagos P.G. (2013). Genetic model selection in genome-wide association studies: robust methods and the use of meta-analysis. Stat. Appl. Genet. Mol. Biol..

[bb0005] Marian A.J. (2012). Molecular genetic studies of complex phenotypes. Transl. Res..

[bb0010] Pritchard J.K., Cox N.J. (2002). The allelic architecture of human disease genes: common disease–common variant…or not?. Hum. Mol. Genet..

[bb0015] Thakkinstian A., McElduff P., D'Este C., Duffy D., Attia J. (2005). A method for meta-analysis of molecular association studies. Stat. Med..

